# The role of collective affective commitment in the relationship between work–family conflict and emotional exhaustion among nurses: a multilevel modeling approach

**DOI:** 10.1186/s12912-019-0329-z

**Published:** 2019-02-18

**Authors:** Maura Galletta, Igor Portoghese, Paola Melis, Cesar Ivan Aviles Gonzalez, Gabriele Finco, Ernesto D’Aloja, Paolo Contu, Marcello Campagna

**Affiliations:** 1 Department of Medical Sciences and Public Health, University of Cagliari, SS554 bivio per Sestu, 09042 Cagliari, Monserrato Italy; 2 Anesthesia and Intensive Care Department, University of Cagliari, Cagliari, Italy; 3 Pain Therapy Service, University of Cagliari, Cagliari, Italy

**Keywords:** Emotional exhaustion, Multilevel analysis, Nurses, Team affective commitment, Work–family conflict

## Abstract

**Background:**

Work–family conflict (WFC) is a crucial problem in nursing because of the demanding conditions of the job, such as strenuous shifts, physical and emotional workload, and intense patient involvement. Using a multilevel approach, this study investigated the moderating role of collective affective commitment as a protective resource in the relationship between WFC and emotional exhaustion.

**Methods:**

The sample included 647 nurses from 66 working units in 4 Italian hospitals. A self-administrated questionnaire was administered to nurses. To analyze data, hierarchical linear modeling was used to examine cross-level relationships between variables.

**Results:**

The results indicated that emotional exhaustion increased with augmenting of WFC and that this relationship was stronger when collective affective commitment was low and weaker when it was high.

**Conclusions:**

The study thus suggests that collective affective commitment may be considered a protective resource for nurses. Moreover, the results show that high work–family conflict should not represent a serious problem when nurses have high affective commitment. Interventions at both individual and group level are discussed in order to mitigate WFC, promoting collective affective commitment and thus reducing emotional exhaustion.

## Background

Work–family conflict (WFC) issues in nurses are inevitable because of the demanding conditions of the job, such as strenuous shifts, physical and emotional workload, and intense patient involvement [1–3]. Furthermore, the increasing prevalence of dual-career couples and single-parent families are social changes that hinder nurses (of all genders) in balancing work and family life [4].

Greenhaus and Beutell [5] defined WFC as “a form of inter-role conflict in which the role pressures and responsibilities from the work and family domains are mutually incompatible so that participation in one role makes it difficult to participate in the other” (p. 77). Based on this definition, studies classify two types of conflict between work and family: *work–family conflict* happens when the pressures of work interfere with the responsibilities of family life, while *family–work conflict* occurs when family life interferes with work responsibilities [6]. Imbalance between working life and private life is considered one of the main stressors in the workplace [7], and available literature shows high proportions of WFC among nurses [8] given the increasing and inescapable demands of their jobs and work conditions; thus, investigating work–family conflict in the nursing profession is important for its clinical practice implications.

Recent studies in nursing show that WFC is associated with turnover intentions [9], depressive symptoms [10], and both job and life dissatisfaction [11]. These effects can be explained using Conservation of Resources theory [12], an integrated model of stress theories, which states that individuals try to attain and maintain resources to get desired outcomes and that stress takes place when there is a feeling of loss (e.g., energy, time, self-efficacy). In this sense, work–family conflict would influence stress, because in WFC resources are lost to struggle to manage and balance work and family roles [12]. Studies show associations between WFC and burnout syndrome—especially its emotional exhaustion component—in health professions [13, 14]. Nursing work is characterized by several risk factors for developing work–family conflict (e.g., shift work, long working hours, responsibility for patients) [15]. Given the nature of nurses’ work, we can expect to find a relationship between high WFC and emotional exhaustion.

### WFC and emotional exhaustion

Burnout is a syndrome characterized by emotional exhaustion, cynicism, and professional inefficacy. Emotional exhaustion—“feelings of being overextended and depleted of one’s emotional and physical resources” [16]— is a consequence of recurrent emotional and physical stress; it is the core element of burnout and is considered to be the first dimension leading to burnout syndrome [16]. At the same time, emotional exhaustion reduces workers’ initiative and progressively limits their capacity to do demanding work [17]. This in turn may lead nurses to become detached from work, developing cynical attitudes and behaviors about their work and patients in response to the overload of emotional exhaustion. This cynicism can in turn reduce their sense of self-esteem and personal effectiveness (and/through their own perceptions of ineffectiveness and lack of productivity at work) [18].

According to the job demand–resource model (JD-R [19]), emotional exhaustion occurs when there is an imbalance in the relationship between job demands and job resources. Job demands include organizational, social, and physical factors that require the individual to use continuous coping strategies. Time, energy, and attention can be limited when people face multiple roles both at work and at home. Based on this model, WFC can be viewed as a kind of demand on both the work and family domains and is likely to be associated with emotional exhaustion [20], thus threatening the quality of the nurse–patient relationship as well as the nurse’s own wellbeing. Negative effects of emotional exhaustion may include absenteeism, poor job performance, mental diseases, anxiety, and job-related injuries [21, 22], all of which can themselves worsen quality of nursing practice, thus threatening the patient’s health [23]. From this theoretical perspective, in this study we focused on emotional exhaustion as a key source of organizational disorder, and hypothesized that:

- *Hypothesis 1*

Work–family conflict is positively related to emotional exhaustion.

### Collective affective commitment as a resource

Job resources, as a counterpart to demands, may be drawn from organizational, social, psychological, and physical aspects of the job and are important to decrease job demands [19]. Working in a satisfying work environment is an important condition for fostering loyal and committed nurses and improving quality of care [24, 25]. Workgroup commitment in particular appears to be a salient form of commitment among nurses, due to the importance of teamwork to patient care (e.g., [26]). Affective commitment is a positive work attitude that can become a resource for nurses due to its strong relationship with well-being (e.g., [27]). In the present study, we focused on collective affective commitment to the workgroup.

Commitment can be defined as a psychological force connecting an individual to a course of action of relevance to one or more targets [28]. This bond can involve different mindsets, such as affective attachment and involvement with the target (affective commitment), felt obligation to the target (normative commitment), or perceived cost associated with interrupting involvement with the target. These three components reflect different types of attachment, and affective commitment in particular contributes to positive results such as increased job satisfaction and well-being among nurses (e.g., [29]).

A workgroup in our context is a collection of individuals embedded in an organizational context who share goals and interact to execute interdependent tasks [30]. Collective affective commitment refers to a shared mindset among a collective of nurses regarding their team/ward, patient care, or a specific task at hand, and is characterized by feelings of loyalty and a desire to invest both mental and physical energy in achieving goals [31, 32]. Research shows that work–family conflict is related to low employee commitment (e.g., [33]); however, the cross-level interaction between WFC and collective affective commitment on the relationship with emotional exhaustion has not been investigated. Affective commitment is strengthened by work experiences that contribute to nurses’ sense of comfort in the workgroup [34]. Unlike normative and continuance commitment, affective commitment gives individuals a sense of belonging, stability, and security [28], and it is likely that this shared bond makes nurses more resistant to stressors or mitigates adverse effects. In a similar vein, collective affective commitment is also hypothesized to be a protective resource: we propose that in a work environment characterized by collective affective commitment, nurses feel emotionally bonded to their workgroup and are more likely to internalize the values and goals of the group, thus reducing the magnifying effects of WFC on emotional exhaustion.

- *Hypothesis 2*

Collective affective commitment moderates the relationship between work–family conflict and emotional exhaustion such that the relationship is weaker when collective affective commitment is higher.

**Fig. 1:**
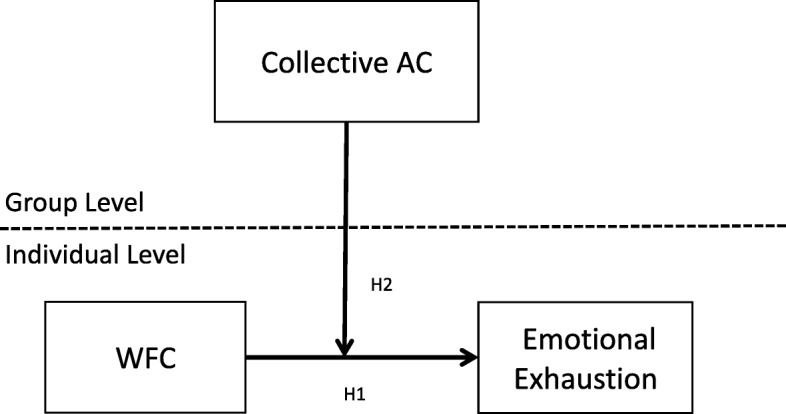
Hypothesized model. AC = affective commitment. WFC = work-family conflict. H = hypothesis

Hence, the aim of the study was to test a multilevel conceptual model of job burnout, with emphasis on how WFC and collective affective commitment function as antecedents of individual experiences of emotional exhaustion. In recent years, in nursing and healthcare contexts, there have been a growing number of scholars who consider the working team as a level of analysis in occupational studies (e.g., [35–37]); in this context, our study provides additional value by investigating the cross-level interaction between WFC and collective affective commitment on the relationship with individual emotional exhaustion (Fig. 1).

## Methods

### Procedure

The sample for the current study consisted of 647 nurses from 66 working units in 4 Italian Hospitals. A self-administrated questionnaire that included scales to measure WFC, emotional exhaustion, affective commitment, and demographic characteristics were distributed to all nurses. Verbal information about the study purpose was given to the participants during programmed meetings. Also, a cover letter accompanying the questionnaire informed about the objectives of the study and assured nurses that their responses were confidential. Participation was voluntary and anonymous. The completed questionnaires were deposited in locked boxes placed in each work unit. Overall, 688 of 1156 questionnaires were returned (response rate of 59.5%). Of these, we removed 41 questionnaires with missing responses for variables relevant to this study.

### Ethical considerations

As this research included nurses’ perception data (through questionnaires), rather than data from patient surveys or therapeutic medication, no formal ethics approval was necessary for the study. In Italy ethical approval is not required for observational nature studies as they are not defined as medical\clinical research, referring to the Italian law 211/2003. However, this study complies with the Declaration of Helsinki in 1995 (as revised in Edinburgh 2000) and with Italian privacy law (Decree n. 196/2003).

Hospital administrations authorized the research. After approval, authorization for administering the questionnaire to the nurses was obtained by ward managers and nurse heads. Written and oral information about the study purpose and modality was provided to the participants by the researchers during programmed meetings. The participation in the study was voluntary and anonymous and data were treated only for scientific purpose. Nurses were also informed about the right to interrupt their participation at any time without consequences for their job. The questionnaires were distributed to nurses by the researchers during working hours. Participants were asked to place the questionnaires in locked boxes once they were completed. Because of the anonymity, written consent was not legally required, nor was it considered necessary. Returning the completed questionnaire was considered as informed consent.

### Measures

The instrument included scales validated internationally. Translation/back-translation procedure [38] was performed for the scales that did not have an Italian validation. Original scales were translated from English into Italian by one bilingual expert and one Italian academic nurse, independently. The translations were compared to adapt sentences linguistically and culturally. After reviewing, the obtained version was back-translated into English by another bilingual linguistic expert to assess the equivalence. After minimal changes, concepts and meanings were considered highly equivalent. To assess appropriateness of cultural adaptation and feasibility of the final version, a pre-test was performed by involving 15 nurses. After pre-test, minimal changes were made to wording.

WFC was measured using five-item scale developed by Netemeyer et al. [6] (eg: “*The demands of my work interfere with my home and my family life*”). Items were answered on a 5-point Likert scale from 1 (*strongly disagree*) to 5 (*strongly agree*). Cronbach’s Alpha coefficient was .91.

We adapted three items from the affective commitment subscale of the Italian validation [39] of the original questionnaire by Allen and Meyer [40]. To increase the relevance to the health sector, ‘ward/group’ was substituted for ‘organization’ in the original scale (eg.: “*This ward/group has a great deal of personal meaning for me*”). Items answered on a 5-point Likert scale from 1 (*strongly disagree*) to 5 (*strongly agree*). Cronbach’s Alpha coefficient was .89.

The emotional exhaustion (4 items) subscale of the Maslach Burnout Inventory-General Survey [41] was used (eg.: “*I feel used up at the end of the work day”*). Items were answered on a 7-point scale from 0 (*never*) to 6 (*every day*). Cronbach’s Alpha coefficient was .77.

#### Control variables

According to Cropanzano, Rupp and Byrne [42], as demographic groups may be differentially impacted by emotional exhaustion, we controlled for gender and unit tenure at Level 1.

### Construct validity analysis

To further examine the distinctiveness of the scales used in this study, we conducted confirmatory factor analysis (CFA) with AMOS (IBM Corp, Armonk, NY). More specifically, we compared the fit of a model in which the measures of all three factors (WFC, collective affective commitment, and emotional exhaustion) were set to load on their respective factors with the fit of more constrained models in which some factors (e.g., WFC and collective affective commitment) were set to load on a single factor.

Because the χ2 statistic is sensitive to sample size, comparison of fit for the three-factor, two-factor, and one-factor solutions was based on the following goodness of fit indices [43]: Tucker Lewis Index (TLI), Comparative Fit Index (CFI) and Root-Mean-Square Error of Approximation (RMSEA). To indicate a good fit of the model, the TLI and CFI critical values should be ≥.90 and RMSEA ≤.08. Finally, to evaluate the two competing models against each other, the χ2 - difference test was used. A non-significant value of this statistic suggests that the overall fit of two models is comparable [44]. The principle of parsimony then suggests that the model with the highest degree of freedom (the most parsimonious model) is the better choice [45].

### Analysis strategy

Given the multilevel nature of the data with nurses were nested in working units, Hierarchical linear Modeling (HLM 6.08) software [46] using the restricted maximum-likelihood estimation method was used to test all the hypotheses. Multilevel modeling is a statistical method that allows researchers to examine cross-level relationships and simultaneously decompose the variances of the study variables into within-group and between-group components [46]. We followed Enders and Tofighi’s recommendations for centering Level 1 and Level 2 variables. Specifically, the Level 1 predictor (i.e., WFC) was centered at its grand mean [47]. For analyses focusing on testing cross-level interaction effect (Hypothesis 2), the Level 1 variable was centered at the mean of each work unit and Level 2 was centered at the grand mean. This approach “yields a pure estimate of the moderating influence that a level 2 predictor exerts on the level 1 association between X and Y” (p. 133) [47].

### Aggregation procedure

We conceptualized affective commitment to the ward at the group level (collective affective commitment). To meet the conditions for multilevel analysis, the assumptions of justifiable aggregation and significant between-group variance were tested. First, rwg(j) scores using a uniform null distribution were used to assess the within-group agreement [48]. The mean rwg(j) was .70. We also assessed intraclass correlation coefficients [ICC(1) and ICC(2)]. Specifically, ICC(1) is the proportion of variance in individual responses that is accounted for by unit membership [49]. It is equivalent to a one-way ANOVA [50], and allows one to partition the within-group and between-group variances. It can be computed using the following formula [50, 51]:

$$ \mathrm{ICC}={\uptau}_{00}/\left({\uptau}_{00}+{\upsigma}^2\right) $$

where, τ_00_ is the between group variance component of the construct and σ^2^ is the within group variance component of the construct. ICC(2) is a measure of the reliability of the unit scores, or the extent to which units can be reliably differentiated. In this sense, it can be interpreted in a similar fashion to other reliability measures. Results showed that ICC(1) was .15. Bliese [49] indicated that ICC(1) values are typically in the range 0.05–0.20. ICC(2) was .63, higher than the recommended cut-off value of 0.60.

### Statistical analyses

As a preliminary analysis, we tested an unconditional model (null model) to estimate the total systematic variance in the outcome variable (essentially one-way analysis of variance estimating the within- and between-groups variance). The proportion of the between-groups variance to the total variance is the ICC(1) value for the dependent variable, providing an assessment of group-level influences.

Then, a two-level random intercepts regression model was constructed with 647 employees (at level 1) nested within 66 work units. First, we regressed emotional exhaustion on the individual-level independent variable (i.e. WFC) in the equation. Significance for the independent variable in such regression equation was an indication of support for the individual-level prediction (i.e. Hypothesis 1). Second, we examined the between-group variance in the slopes of the relationship between the individual-level independent variables and emotional exhaustion. Significant between-group variance in the slopes of these relationships was an indication of the presence of possible moderator at the group level. Third, we introduced collective affective commitment as a level 2 moderator of the level 1 relationship. Significance for the interaction term involving collective affective commitment was an indication of support for our cross-level prediction (i.e., Hypothesis 2). Fourth, we plotted the significant interaction at two levels of collective affective commitment (i.e., + 1 SD and − 1 SD) [52] and conducted simple slopes tests to examine the nature of the interactions. Finally, given that the testing of moderator-hypothesis requires the inclusion of all main effects in the regression equation [52], we controlled for the main effects of affective commitment at level 2 when testing for the interaction term.

Specifically, the equations for our hypotheses are:

- *Hypothesis 1*:
  - Level 1: Emotional Exhaustion_ij_ = β_0j_ + β_1j_^∗^(WFC_ij_) + e_ij_
  - Level 2: $$ {\displaystyle \begin{array}{c}{\upbeta}_{0\mathrm{j}}={\upgamma}_{00}+{\mathrm{U}}_0\\ {}{\upbeta}_{1\mathrm{j}}={\upgamma}_{10}+{\mathrm{U}}_1\end{array}} $$
- *Hypothesis 2*:
  - Level 1: Emotional Exhaustion_ij_ = β_0j_ + β_1j_^∗^(WFC_ij_) + e_ij_
  - Level 2: $$ {\displaystyle \begin{array}{c}{\upbeta}_{0\mathrm{j}}={\upgamma}_{00}+{\upgamma_{01}}^{\ast}\left(\mathrm{Collective}\ \mathrm{Affective}\ \mathrm{Commitment}\right)+{\mathrm{U}}_{0\mathrm{j}}\\ {}{\upbeta}_{1\mathrm{j}}={\upgamma}_{10}+{\upgamma_{11}}^{\ast}\left(\mathrm{Collective}\ \mathrm{Affective}\ \mathrm{Commitment}\right)+{\mathrm{U}}_{1\mathrm{j}}\end{array}} $$

## Results

### Characteristics of participants

The data from 647 nurses working in 66 units were used for the analysis. The majority of respondents (58.9%) were women. The proportion of nurses aged 47–55 years was the highest (39.7%), 24.9% aged 40–46 years, and 16.7% aged over 55 years, and the remaining 18.7% aged less than 40 years. With regard tenure in the work unit, 46.7% of nurses worked in the same ward for more than 10 years.

Table 1 presents means, standard deviations and Pearson correlations of the variables studied in this research.

**Table 1:** Means, standard deviations and Pearson correlations of the study variables (*n* = 647)

	M	SD	1	2	3
1. Emotional exhaustion	2.65	1.43	(.77)		
2. Work-family conflict	2.84	.98	.45^a^	(.91)	
3. Collective affective commitment	3.67	.47	-.40^a^	-.27^a^	(.87)

*Note*. ^a^Correlation is significant at the .01 level

### Confirmatory factor analysis

The results for CFA revealed that the hypothesized three-factor model fitted the data: χ2 = 420.49 df = 51, *p* < .001, RMSEA = .11, CFI = .90, TLI = .84. However, inspection of modification indices and standardized residuals suggested that model fit could be improved if correlated error was estimated. Thus, the three-factor model was refitted to the data allowing for error correlation between four couple of errors. Fit indices for the revised model indicated improved fit: χ2 = 183.36 df = 47, *p* < .001, RMSEA = .07, CFI = .96, TLI = .94. This last model fitted the data significantly better than the one-factor model (χ2 = 1815.64, df = 54, p < .001, RMSEA = .23, CFI = .50, TLI = .28), providing evidence for the convergent and discriminant validity of the measurement model variables.

### Multilevel analysis

Table 2 shows the results of multilevel regression analyses.

**Table 2:** HLM analysis with emotional exhaustion as dependent variable

Effect	Unconditional model		Model 1		Model 2	
	Estimate	se	Estimate	se	Estimate	se
Level 1:						
Intercept (γ_00_)	2.62	.08	2.62	.08	2.63	.07
Gender			−.16	.10	−.05	.10
Unit tenure			.10	.06	.10	.06
WFC (γ_10)_			.63**	.07	.62**	.06
Level 2:						
Cross-level interaction effects						
Collective AC (γ_11)_					-. 28*	.13
Variance components						
Within-team (L1) variance (σ^2^)	1.80		1.40		1.40	
Intercept (L2) variance (τ00)	.24		.29		.17	
Slope (L2) variance (τ11)			.08		.08	

*Note*. Individuals, n 647; Teams, n 66. (Unstandardized regression coefficients). *WFC* work-family conflict, *AC* affective commitment

**p < .001, *p < .01. In Level 1 analysis, WFC was group-mean centered. Gender and unit tenure were not centered

Before testing our hypotheses, we inspected the result of the null model in HLM. The results showed that ICC(1) for emotional exhaustion was .24, F(64) = 148.48, p < .001, which indicates that 24% of the variance in emotional exhaustion was due to work unit membership. These results confirmed that emotional exhaustion varied significantly between nurses as well as across working units, thus justifying the use of HLM for further inspection of multilevel variables explaining the variation in emotional exhaustion.

#### Effect of WFC on emotional exhaustion

Hypothesis 1 posited that WFC would be positively associated with emotional exhaustion.

At level 1, after controlling for gender and unit tenure, we regressed emotional exhaustion on WFC (see Model 1, Table 2). Results indicated that gender and unit tenure were not significant, and WFC was positively related to emotional exhaustion (γ_10_ = .63, *p* < .01). Hence, Hypothesis 1 was supported.

#### Cross-level interaction effect of collective affective commitment on the relationship between WFC and emotional exhaustion

**Fig. 2:**
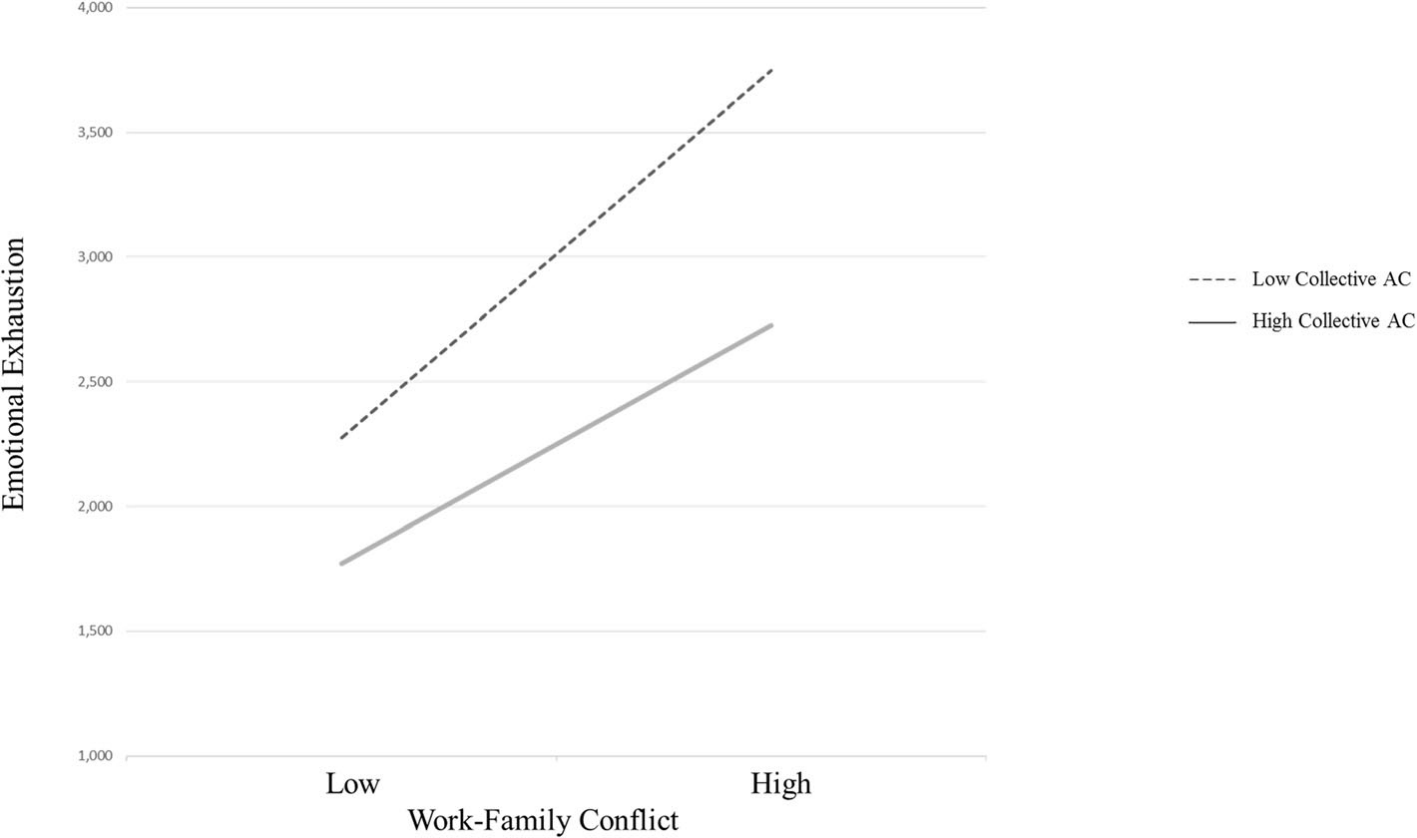
Cross-level interaction. Moderation of collective affective commitment on the relationship between work-family conflict and emotional exhaustion. AC = affective commitment

Next, we examined the moderating effect of collective affective commitment (see Model 2, Table 2) using the steps described above. According to Hypothesis 2, the relationship between WFC and emotional exhaustion is moderated by collective affective commitment such that the relationship is stronger when collective affective commitment is lower. Results showed a significant effect for collective affective commitment as a level 2 (i.e., group-level) moderator (γ_11_ = −.28, *p* < .05). This interaction is graphically represented in Fig. 2.

A simple slopes test indicated that WFC was related to emotional exhaustion at both lower (γ = .75, p < .01) and higher levels of collective AC (γ = .49, p < .01). Hypothesis 2 was therefore supported.

## Discussion

As per its aim, this study tested a multilevel model of job burnout and highlighted how WFC and collective affective commitment function as antecedents of individual experiences of emotional exhaustion. As part of this investigation, we provided support at the individual level for the positive relationship between WFC and emotional exhaustion, in line with Leineweber et al. [14].

In the JD-R model, role stressors such as WFC are an important aspect of job demands and a source of reduced well-being among workers. Our results support previous research in which WFC has shown a direct association with emotional exhaustion [13, 14]. When a work environment provides poor opportunity to balancing between work and personal life, this can generate uncertainty about what behavior is desirable [53]. In the nursing context, work characteristics such as shift work and night shifts, long work hours per week, responsibility for others’ health and safety, workload, and job emotional involvement represent a risk of work–family conflict [15, 54]. The results of this study suggest that WFC stems from demands on both the work and family domains, which can threaten nurses’ health (in particular, by leading to emotional exhaustion). This is important because literature shows that nurse burnout can worsen the quality of practice, thus compromising patient’s health [23]. Therefore, it is crucial to plan actions aimed at limiting the imbalance between working and family roles among nurses. In the Italian context, investigated here, Italian law 81/2008 requires the evaluation of psychosocial risks to safeguard workers’ psychological well-being, constituting another reason for such planning [55]. Management-level support actions to meet nurses ‘needs are the most effective in reducing WFC [54] and may to reduce psychosocial risk, thus potentially improving collaborative team-based care.

Another main result of this study relates to the moderating role of collective affective commitment in the effect of WFC on emotional exhaustion. Research indicated that more knowledge is needed on processes buffering occupational stress. Taris [56] highlighted that, of the 90 studies testing a buffering effect, only 10% provided support for the interaction effect. The results of this research showed a relationship between work–family conflict and emotional exhaustion: when the clash between work and family duties increases, emotional exhaustion increases, and that this relationship is stronger when collective affective commitment is low and weaker when it is high. Thus, following Meyer and Herscovitch [28], this study suggests that nurses’ sense of collective belonging to the workgroup plays an important role in making them more resistant to stressors, thus protecting them from emotional exhaustion when work–family imbalance increases. This study thus suggests that collective affective commitment may be considered a protective resource for nurses. Moreover, the results show that high work–family conflict should not represent a serious problem when nurses have high affective commitment, which should occur when they share a work environment that makes them feel safe, stable, and desirous to invest mental and physical energy in achieving organizational goals. As burnout develops mainly in the social context of the workplace and is characterized by ongoing individual perception, interpretation, and construction of others’ workplace behaviors [57], these findings provide support for the JD-R model’s assumption that interpersonal job resources (specifically, collective affective commitment) may act as a buffer in the relationship between job demands (represented by WFC) and job burnout (represented by emotional exhaustion).

### Limitations

This study has some limitation that should be addressed. First, our study involved units in only four hospitals. Involving a larger number of hospitals would allow exploration of both hospital- and unit-level effects using multilevel techniques. Second, we used cross-sectional data and therefore are not able to explain the causal influence [58] of the relationship between variables. To enhance the generalizability of our study, future research should employ longitudinal designs to investigate the long-term effects of collective affective commitment and WFC on burnout behavior; this study has investigated only emotional exhaustion, considered as the first dimension leading to the syndrome [16]. Despite these limitations, this study is one of the first concerning the relationship between WFC, collective affective commitment, and emotional exhaustion at different organizational levels, and thus makes a novel contribution to the growth of knowledge in this important nursing management area.

### Implications for nursing practice

One of the most interesting practical implications of this study is that enhancing collective affective commitment and decreasing work–family conflict can result in reduced emotional exhaustion at the individual level.

The multilevel perspective allows interventions focusing on both the individual and group levels [59]. At the individual level, interventions based on management and prevention should be implemented to mitigate work–family imbalance and reduce emotional exhaustion. Effective interventions could include allowing self-scheduling of work shifts, implementing flexible work schedules, clarifying task division to avoid excessive workload, and replacing absent personnel. External opportunities could involve time-saving solutions such as agreements with laundries and supermarkets, etc., provision of recreational activities, and provision of mental health and counseling support for nurses [60]. Also, continuing education for head nurses should train them to become adequate mediators between nurses and doctors, thus preventing excessive request by the latter of the former [61]. However, it is important that these interventions reflect the actual nurses’ needs and are based on nurses’ input.

At the group level, interventions should be based on promoting collective affective commitment. Hospital wards can increase affective commitment of their nurses by promoting both supervisor and organizational support [62, 63]. Practices that enhance motivation and empowerment can also play an important role in improving collective commitment [31] and thus reducing individual emotional exhaustion. In addition, training at the team level should include a focus on fostering team collaboration, team identification, and supportive team climate. Considering our results regarding the strong effect of collective affective commitment, increasing this resource among nurses may likely decrease burnout risk, and in turn, promote healthy workplaces.

## Conclusion

The aim of this study was to expand the JD-R Model beyond the individual level of analysis, while emphasizing the active role of collective affective commitment as a protective resource against emotional exhaustion. In general, the multilevel approach provided additional value in examining burnout risk among nurses who share similar experiences and feelings within the same working context.

## Abbreviations

CFA: Confirmatory factor analysis
CFI: Comparative Fit Index
HLM: Hierarchical linear Modeling
ICC: Intraclass correlation coefficient
JD-R: Job demand-resource model
RMSEA: Root-Mean-Square Error of Approximation
TLI: Tucker Lewis Index
WFC: Work-family conflict
